# A New Intrusion Detection System for the Internet of Things via Deep Convolutional Neural Network and Feature Engineering

**DOI:** 10.3390/s22103607

**Published:** 2022-05-10

**Authors:** Safi Ullah, Jawad Ahmad, Muazzam A. Khan, Eman H. Alkhammash, Myriam Hadjouni, Yazeed Yasin Ghadi, Faisal Saeed, Nikolaos Pitropakis

**Affiliations:** 1 Department of Computer Science, Quaid-i-Azam University, Islamabad 44000, Pakistan; safiullah@cs.qau.edu.pk (S.U.); muazzam.khattak@qau.edu.pk (M.A.K.); 2 School of Computing, Edinburgh Napier University, Edinburgh EH10 5DT, UK; n.pitropakis@napier.ac.uk; 3 Pakistan Academy of Sciences, Islamabad 44000, Pakistan; 4 Department of Computer Science, College of Computers and Information Technology, Taif University, P.O. Box 11099, Taif 21944, Saudi Arabia; eman.kms@tu.edu.sa; 5 Department of Computer Sciences, College of Computer and Information Science, Princess Nourah Bint Abdulrahman University, P.O. Box 84428, Riyadh 11671, Saudi Arabia; mfhaojouni@pnu.edu.sa; 6 Department of Computer Science and Software Engineering, Al Ain University, Abu Dhabi 122612, United Arab Emirates; yazeed.ghadi@aau.ac.ae; 7 DAAI Research Group, Department of Computing and Data Science, School of Computing and Digital Technology, Birmingham City University, Birmingham B4 7XG, UK; faisal.saeed@bcu.ac.uk

**Keywords:** convolution neural network, cybersecurity, deep learning, Internet of Things, intrusion detection

## Abstract

The Internet of Things (IoT) is a widely used technology in automated network systems across the world. The impact of the IoT on different industries has occurred in recent years. Many IoT nodes collect, store, and process personal data, which is an ideal target for attackers. Several researchers have worked on this problem and have presented many intrusion detection systems (IDSs). The existing system has difficulties in improving performance and identifying subcategories of cyberattacks. This paper proposes a deep-convolutional-neural-network (DCNN)-based IDS. A DCNN consists of two convolutional layers and three fully connected dense layers. The proposed model aims to improve performance and reduce computational power. Experiments were conducted utilizing the IoTID20 dataset. The performance analysis of the proposed model was carried out with several metrics, such as accuracy, precision, recall, and F1-score. A number of optimization techniques were applied to the proposed model in which Adam, AdaMax, and Nadam performance was optimum. In addition, the proposed model was compared with various advanced deep learning (DL) and traditional machine learning (ML) techniques. All experimental analysis indicates that the accuracy of the proposed approach is high and more robust than existing DL-based algorithms.

## 1. Introduction

**Figure 1:**
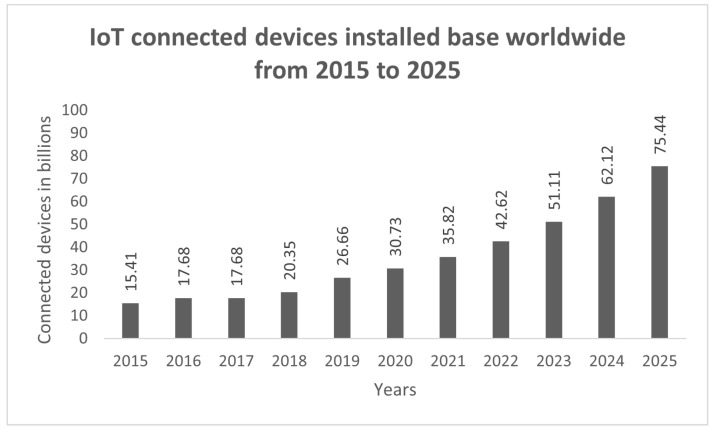
Growth of IoT devices from 2015 to 2025 [5].

The IoT foresees the networking of a wide range of smart things in our environment that are capable of accumulating, processing, and communicating data [1]. The IoT is a widely used technology in automated network systems across the world that has had an impact on different areas, such as the agricultural, medical, transport, and automobile industries, and water monitoring in recent years [2,3]. The use of IoT devices has increased dramatically, from 15.41 billion in 2015 to more than 35.8 billion in 2021, as homes and businesses increasingly rely on online technology [4]. The IoT is anticipated to reach 75.44 billion devices by 2025, as shown in Figure 1, which will generate 79 zettabytes (ZB) of data [5]. The IoT has been identified as a critical component of digitization for a transforming society [6].

Many IoT devices capture, store, and process personal data, making them a feasible target for assailants because of their distributed structure and openness [7]. The effective deployment of IoT networks is becoming more dependent on security [8]. An IDS is required to examine IoT network traffic for the identification of cyberattacks [9]. Several researchers have worked on IDSs in which machine learning (ML) and deep learning (DL) models play a key role [10]. ML and DL techniques are widely used in different fields, such as in agriculture [11], medical [12], and automobile industries [13,14]. DL is a branch of ML, and it is generalizable to new problems with complicated and high-dimensional data. Furthermore, DL methods allow for the training of nonlinear models on big datasets in a systematic way [15]. This is why DL performs well in detecting intrusions, as it not only handles a large amount of data but also can generalize to new types of attacks in the network [16].

The existing system has difficulties in improving performance and identifying subcategories of cyberattacks. This paper proposes a DCNN followed by a deep-neural-networks (DNN)-based IDS. The primary advantage of a DCNN is its ability to exploit the correlation between features [17]. A DCNN works on a lower number of parameters than other DL models [18]. Thus, the required computational power is decreased, and the learning process is improved. The proposed system improves the performance of existing IDSs and extends to subcategories of malicious attack detection in IoT networks. The IoT network intrusion dataset 2020 (IoTID20) was used for experiments on the proposed model. This dataset includes data for binary, multi-category, and subcategories of IoT networks.

### Contributions

- We proposed a DCNN technique for malicious activity identification in IoT networks.
- We improved performance and reduced the computational power of an IDS for low-power IoT devices in the network.
- We identified the subcategory of cyberattacks in the IoT networks.
- We compared the proposed scheme with other DL and traditional ML techniques.

The remainder of the article is organized as follows. Section 2 discusses related work and presents a literature comparison. A step-by-step methodology of the proposed system is presented in Section 3. Section 4 provides a detailed analysis of the results and a comparison with state-of-the-art models. This work is concluded in Section 5.

## 2. Related Works

Security is an essential part of an IoT network for stability, reliability, and safe communication. Several researchers have proposed different techniques for the detection of malicious attacks in IoT networks. Basati et al. [19] presented an IDS called deep feature extraction (DFE). This model is based on a CNN. The authors mainly focused on those devices that have low processing power. They used UNSW-NB15, CICIDS2017, and KDDCup99 datasets for their experiments. The model was tested for both binary and multi-class classifications. Rashid et al. [20] proposed a stacking ensemble approach based on trees for intrusion detection in the IoT. Two incursion datasets, NSL-KDD and UNSW-NB15, were used to evaluate the efficacy of the proposed model. They also improved efficacy by integrating feature selection strategies to identify the most relevant features.

Fatani et al. [21] introduced a novel feature engineering technique for the IDS system while using the benefits of swarm intelligence (SI) techniques. Four popular public datasets, CIC2017, NSL-KDD, BoT-IoT, and KDD99, were utilized to test the quality of the proposed IDS technique. Alkahtani et al. [22] suggested three advanced and widely used DL models for intrusion detection. The authors conducted experiments on long short-term memory (LSTM), CNN, and a hybrid model of CNN–LSTM. They used the IoTID20 dataset for the evaluation of these DL models. Keserwani et al. [23] presented a method for extracting significant IoT network features for intrusion detection. The proposed method consists of a combination of grey wolf optimization (GWO) and particle swarm optimization (PSO). They utilized the KDDCup99, NSL-KDD, and CICIDS-2017 datasets.

A single hidden layer feedforward neural network (SLFN) method was introduced by Qaddoura et al. [24] for malicious activity detection in IoT networks. The authors used data reduction with clustering and the SMOTE oversampling technique. For the evaluation of the model, they used accuracy, precision, recall, and G-mean. Saba et al. [25] introduced a two-stage hybrid technique for the detection of malicious attacks in IoT networks. A genetic algorithm (GA) was used to choose relevant features as well as the famous ML techniques, such as support vector machine (SVM), ensemble classifier, and decision tree (DT).

The existing systems cannot identify the subcategories of multi-class attacks in the network. In addition, for binary and multi-class detection, the performance of the existing system can be improved. A comparison of the related work is given in Table 1.

## 3. The Proposed Framework

This section provides a detailed explanation of the utilized dataset, preprocessing approaches, the proposed deep convolutional neural network (DCNN), and evaluation metrics.

### 3.1. IoTID20 Dataset

The IoTID20 dataset was developed to identify cyberattacks in IoT networks. This dataset was generated through home-connected smart devices using SKT NGU and EZVIZ Wi-Fi cameras [26]. The main advantage of this dataset is that it includes modern communication data and new data on network interference detection. This dataset has 83 IoT network features and three labels [27]: binary, category, and subcategory; details are given in Table 2.

### 3.2. Preprocessing

Data preprocessing is an essential step for ML/DL methods. Preprocessing converts data into a suitable format for any neural network. This section consists of cleaning, label encoding, feature engineering, normalization, and data splitting.

#### 3.2.1. Dataset Cleaning

A dataset must be verified for empty and undefined instances before training a model. In this experiment, the Python built-in library (Pandas) was used to validate the dataset. The utilized IoTID20 dataset has some missing values. To clean the dataset, we removed all missing value instances.

#### 3.2.2. Label Encoding

Label encoding is a well-known encoding approach for dealing with categorical values. It assigns a unique numeric value to each categorical value. For ML algorithms and DL neural networks to operate, the input and output values must be integers. The utilized dataset has some categorical features. Each categorical feature has several categories for which one-hot encoding requires greater memory and more time [28]. In this study, the label encoder approach was used to convert the categorical features into numeric.

#### 3.2.3. Feature Engineering

Each dataset contains its own set of features. If a dataset contains multiple features as well as certain insignificant features that have no impact on the output label, we must eliminate those features from the dataset because they lead to overfitting and underfitting, which significantly influence the executing time and performance of the classifier. In this study, the filter approach was used. In filtering features, the extra tree classifier (ETC) technique was applied. This method calculates the impact of each feature on the output label. The utilized dataset has 83 features. We select all the features greater than 0.001 for information gain. After applying the feature filtering approach, 62 features were selected.

#### 3.2.4. Normalization

Normalization is a method commonly used in the preprocessing of data for ML/DL algorithms. The purpose of normalization is to convert the numeric column values in a dataset to a common scale while maintaining variations in value ranges. Each feature of the IoTID20 dataset has different values. Some feature values are in the thousands, and some have negative values that reduce the model performance. To solve this problem, the data are normalized between 0 and 1 via min–max method, as represented by Equation (1). Data are converted into an array and reshaped (number of total records, number of input features, 1) using Python’s NumPy library.

(1) $$X_{\mathrm{norm}\;}=\frac{x-x_{\min}}{x_{\max}-x_{\min}}$$

#### 3.2.5. Data Splitting

Splitting the data into train and test sets is one of the common preprocessing steps used to evaluate the ML/DL models’ performance. In an unbalanced dataset, random splitting of datasets can lead to an unequal split of data, which cannot evaluate the performance of the model accurately. To address this problem, we used a stratified method to split the dataset into train and test sets. A stratified sampling procedure splits the entire dataset into homogenous sets. In this work, the stratified method splits the data into 80% train and 20% test sets for each class. A detailed splitting of the cleaned dataset for binary, category, and subcategory classification is given in Table 3.

### 3.3. Designing the DCNN Model

**Figure 2:**
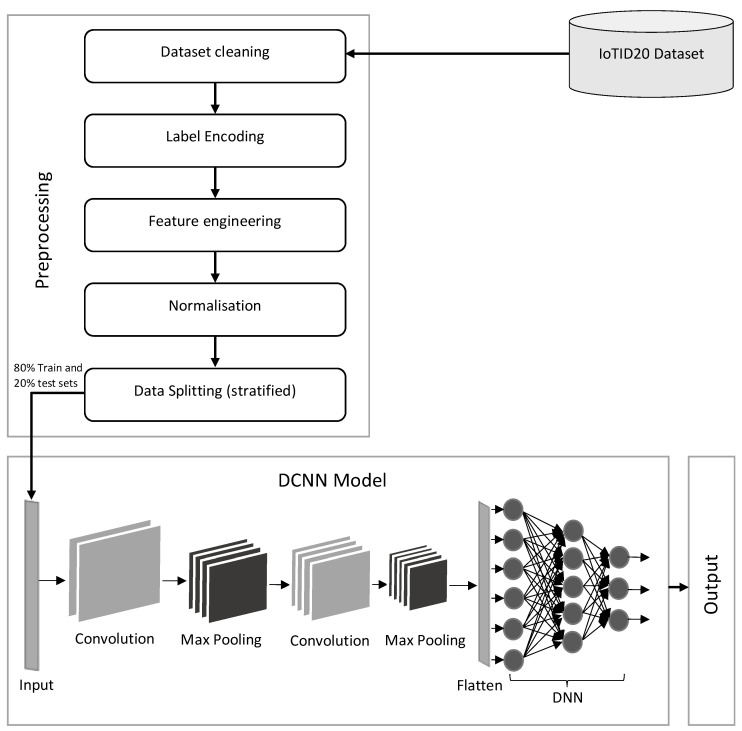
Architecture of the proposed DCNN model.

CNN is a DL technique that consists of convolutional layers, pooling layers, and fully connected layers [29]. CNN is usually utilized for image classification and voice recognition. In this study, we used a DCNN followed by a DNN for malicious activities identification in IoT networks. The proposed approach consists of two 1D convolutional layers, two max-pooling layers, flatten, and three dense layers, as shown in Figure 2. The input shape in the first convolutional layer is (none, 62, 1). Here, “none” is the dynamic number of instances, “62” is the number of input features and “1” is the third-dimension value. The size of the kernel is three, and sixty-two filters were used in this layer, which produces output in the form of (none, 62, 62). The output of the first convolutional layer is given as an input in the max-pooling layer. In this layer, pool size four was used which produces (none, 15, 62) output. The second convolutional layer is placed here, in which the size of the kernel is three and thirty filters are used, which produce the output in the form of (none, 15, 30). The output of the second convolutional layer is given as an input in the max-pooling layer. In this layer, pool size two was used, which produces (none, 7, 30) output. The convolutional layer not only converges the most important features but also reduces noise [30]. The 1D convolutional layer is demonstrated in Equations (2) and (3).

(2) $$x_{k}=b_{k}+\sum_{i=1}^{N}\left(s_{i},w_{ik}\right)$$

(3) $$y_{k}=f\left(x_{k}\right)$$

where $x_{k}$ is the input in the 1D convolutional layer. The output of the previous layer neuron is represented by $s_{k}$, $w_{ik}$ represents the kernel from *i* to *k*. $b_{k}$ is the bias value of the neuron in the convolutional layer. The ReLU activation function is represented by f(). Equation (4) describes the ReLU. $y_{k}$ is the output of the 1D convolutional layer. The output of the convolutional layer is the input in the pooling layer demonstrated in Equation (5). We select the maximum value from region *ℜ* which contains the output values of the convolutional layer. $s_{k}$ is the output of the max-pooling layer.

(4) $$f\left(x_{k}\right)=\max\left(0,x_{k}\right)$$

(5) $$s_{k}{=}_{i\in ℜ}^{\max}y_{k}$$

The flatten method is used to convert the output shape of the last pooling layer into a single-dimensional array. The output of the flatten is (none, 210) which is input in the first dense layers. The output of the first dense layer is (none, 50) which is given as input in the second dense layer. The second dense layer produces (none, 25) output which is input in the last dense layer. The ReLU activation function is used in dense layers. The last dense layer produces output results in which sigmoid function for binary classification and softmax function for multi-class classification are used, respectively. Sigmoid and softmax are demonstrated in Equations (6) and (7).

(6) $$\sigma \left(x\right)=\frac{1}{1+e^{-x}}$$

(7) $$\mathrm{softmax}{\left(x\right)}_{i}=\frac{e^{x_{i}}}{\sum_{j=1}^{K}e^{x_{j}}}$$

### 3.4. Evaluation Metrics

The evaluation of the DCNN approach was carried out with accuracy, precision, recall, and F1-score. We start by explaining these four parameters, true positive (TP), false negative (FN), false positive (FP), and true negative (TN), which are used to compute the evaluation metrics such as accuracy, precision, recall, and the F1-score. TP refers to the number of instances that have been correctly identified as normal. The number of instances that misclassify normal data as an attack is known as the FN. FP represents the number of malicious instances that are wrongly classified as normal. TN represents the number of instances that are classified correctly as malicious. All of these evaluation metrics were calculated by using Equations (8)–(11).

(8) $$\mathrm{Accuracy}=\frac{\alpha +\beta }{\alpha +\beta +\gamma +\delta }$$

(9) $$\mathrm{Precision}=\frac{\alpha }{\alpha +\gamma }$$

(10) $$\mathrm{Recall}=\frac{\alpha }{\alpha +\delta }$$

(11) $$F1\text{-}\mathrm{score}\;=\frac{2\times (\mathrm{Precision}\;\times \;\mathrm{Recall})}{\;\mathrm{Precision}\;+\;\mathrm{Recall}\;}$$

where α represents TP, β represents TN, γ represents FP, and δ represents FN.

### 3.5. Experimental Platform

Experiments on the DCNN model were conducted with the HP ProBook G5 8th generation laptop. This laptop contains 24 GB ram and an Intel Core i5 processor. In software specifications, we used Windows 11 Pro, Python 3.8.5, Tensorflow, and Keras library.

## 4. Performance Analysis

This section provides a detailed evaluation of the proposed model. The proposed DCNN model was evaluated on the IoTID20 dataset. The performance of the DCNN was tested for binary, multi-class categories, and multi-class subcategories classifications. This section presents a comparison of convolutional layers followed by dense layers for multi-class categories and multi-class subcategories. The same comparison was performed for famous optimizers. The optimal solutions were selected from the comparison and compared with other ML/DL models.

### 4.1. Performance Evaluation of Convolutional and Dense Layers

The CNN algorithm consists of convolutional layers, pooling layers, and fully connected layers. This experiment was conducted for one and two convolutional layers, followed by fully connected dense 1–5 layers. These experiments were conducted for the multi-class category and subcategory classification. A detailed comparison is given in Table 4 and Table 5. The experimental results showed that the average optimal solution is two convolutional layers and three dense layers.

### 4.2. Performance Evaluation of Optimizers

An optimizer is a function used to update the neural network weights and learning rates. It helps to reduce the loss and improve the performance of the model [31,32]. Famous optimizers for DL algorithms are stochastic gradient descent (SGD), root mean square propagation (RMSProp), adaptive moment estimation (Adam), adaptive moment estimation maximization (AdaMax), and Nesterov-accelerated adaptive moment estimation (Nadam). The performances of these modifiers are optimal for CNN, as validated in Ref. [33]. The aforementioned five optimizers were used in this experiment. A detailed comparison of optimizers for the multi-class category and subcategory classification is shown in Table 6 and Table 7, respectively. The experimental results show that Adam, Nadam, and AdaMax were the top three optimizers in this experiment.

### 4.3. Performance Analysis of the Proposed DCNN

In this study, we propose a DCNN architecture for malicious activities identification in IoT networks. For DCNN, the above results show that the optimal solution for the IoTID20 dataset is two convolutional layers, followed by three dense layers. In addition, from the above results, we selected the top three optimizers (Adam, Nadam, and AdaMax) for this experiment. This section provides a detailed classification of binary-class, multi-class category, and multi-class subcategories for batch sizes 32, 64, 128, and 256.

#### 4.3.1. DCNN Evaluation for Binary-Class Classification

**Figure 3:**
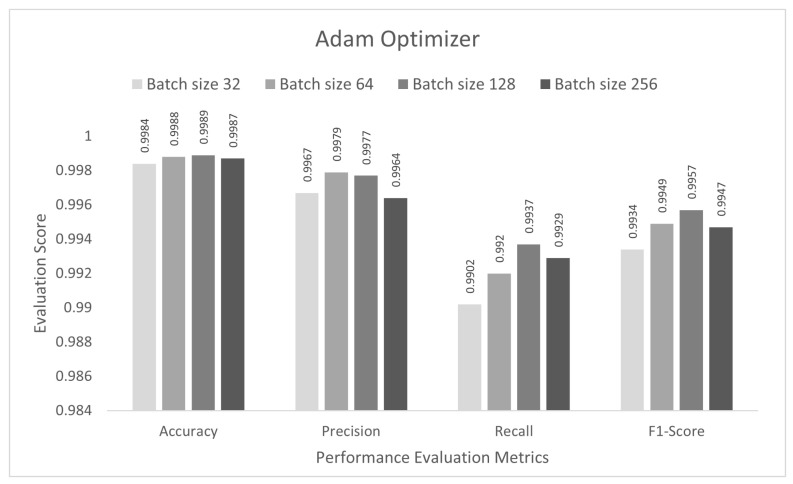
Adam optimizer for binary class scenario.

**Figure 4:**
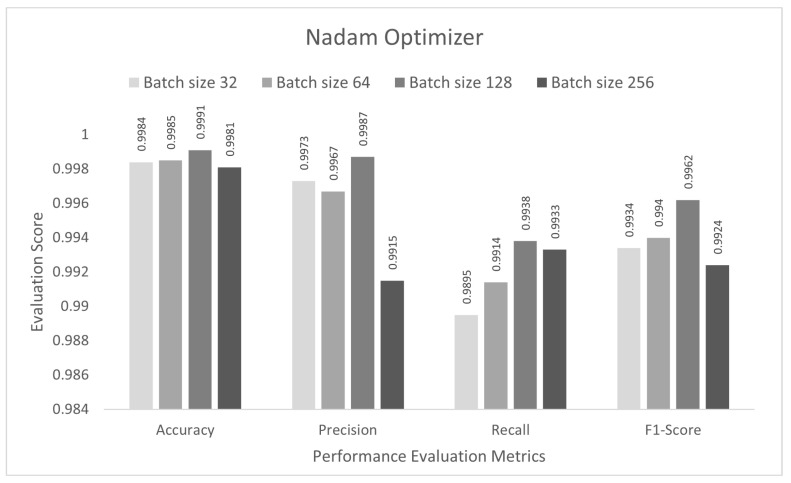
Nadam optimizer for binary class scenario.

**Figure 5:**
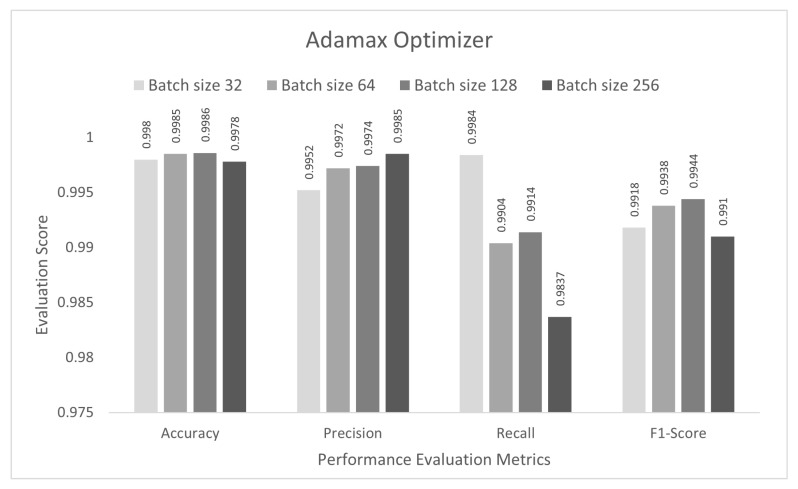
AdaMax optimizer for binary class scenario.

The performance of the proposed approach was tested for a binary-class scenario. The DCNN model was trained with the IoTID20 dataset for 50 epochs, and the binary cross-entropy function was used to calculate the loss. In the first step, the proposed DCNN performance for the Adam optimizer is compared in the bar graphs in Figure 3. Based on the findings, the proposed model had the highest anomaly detection accuracy of 99.89% at batch size 128. For this optimizer, the other evaluation scores, namely, precision, recall, and F1-score, were 99.77%, 99.37%, and 99.57%, respectively. In the second step, all the experiments for the Nadam optimizer were rearranged with the same batch sizes. The proposed DCNN performance for the Nadam optimizer is compared in the bar graphs in Figure 4. Based on the findings, the proposed model had the highest anomaly detection accuracy of 99.91% at batch size 128. For this optimizer, the other evaluation scores, namely, precision, recall, and F1-score, are 99.87%, 99.38%, and 99.62%, respectively. In the third step, all the experiments for the AdaMax optimizer were repeated with the same batch sizes. The proposed DCNN performance for the Nadam optimizer is compared in the bar graphs in Figure 5. Based on the findings, the proposed model had the highest anomaly detection accuracy of 99.86% at batch size 128. For this optimizer, the other evaluation scores, namely, precision, recall, and F1-score, were 99.74%, 99.14%, and 99.44%, respectively.

#### 4.3.2. DCNN Evaluation for Multi-Class Category Classification

**Figure 6:**
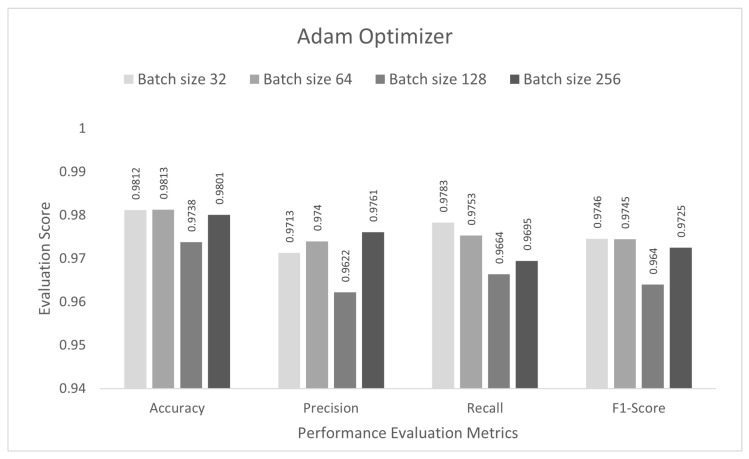
Adam optimizer for multi-class category classification scenario.

**Figure 7:**
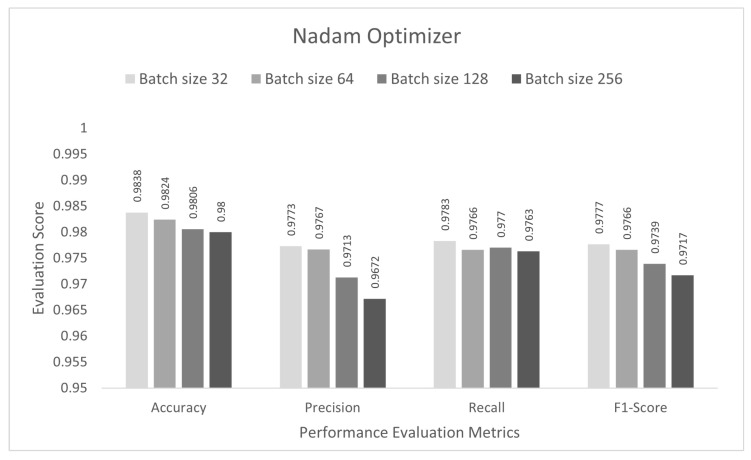
Nadam optimizer for multi-class category classification scenario.

**Figure 8:**
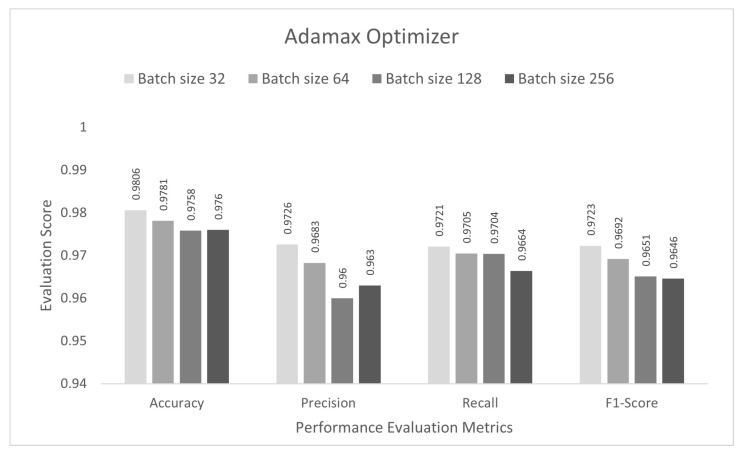
AdaMax optimizer for multi-class category classification scenario.

In this stage, the performance of the proposed study was evaluated for a multi-class category classification scenario. The DCNN model was trained with the IoTID20 dataset for 50 epochs, and a sparse categorical cross-entropy function was used to calculate the loss. As noted previously, for the binary-class studies, an Adam optimizer was chosen at the initial stage. The proposed DCNN performance for the Adam optimizer is compared in the bar graphs in Figure 6. Based on the analysis of the results, the proposed model had the highest anomaly detection accuracy of 98.13% at batch size 64. For this optimizer, the other performance scores, namely, precision, recall, and F1-score, were 97.40%, 97.53%, and 97.45%, respectively. In the second step, all the experiments for the Nadam optimizer were rearranged with the same batch sizes. The proposed DCNN performance for the Nadam optimizer is compared in the bar graphs in Figure 7. Based on the analysis of the results, the proposed model had the highest anomaly detection accuracy of 98.38% at batch size 32. For this optimizer, the other performance scores, namely, precision, recall, and F1-score, were 97.73%, 97.83%, and 97.77%, respectively. In the third step, all the experiments for the AdaMax optimizer were repeated with the same batch sizes. The proposed DCNN performance for the Nadam optimizer is compared in the bar graphs in Figure 8. Based on the analysis of the results, the proposed model had the highest anomaly detection accuracy of 98.06% at batch size 32. For this optimizer, the other performance scores, namely, precision, recall, and F1-score, were 97.26%, 97.21%, and 97.23%, respectively.

#### 4.3.3. DCNN Evaluation for Multi-Class Subcategory Classification

**Figure 9:**
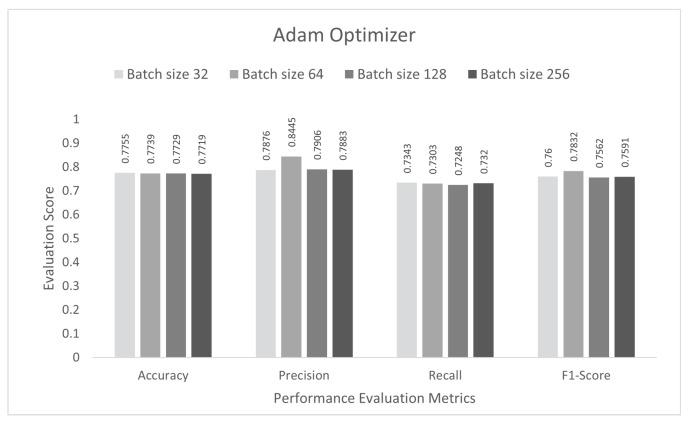
Adam optimizer for multi-class sub-category classification scenario.

**Figure 10:**
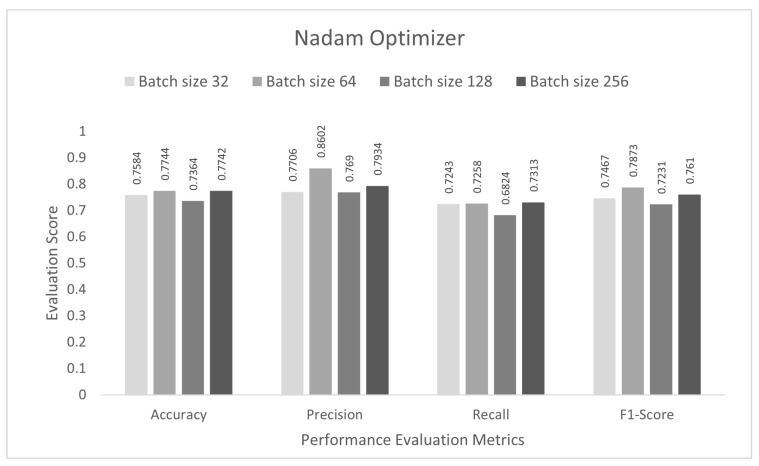
Nadam optimizer for multi-class sub-category classification scenario.

**Figure 11:**
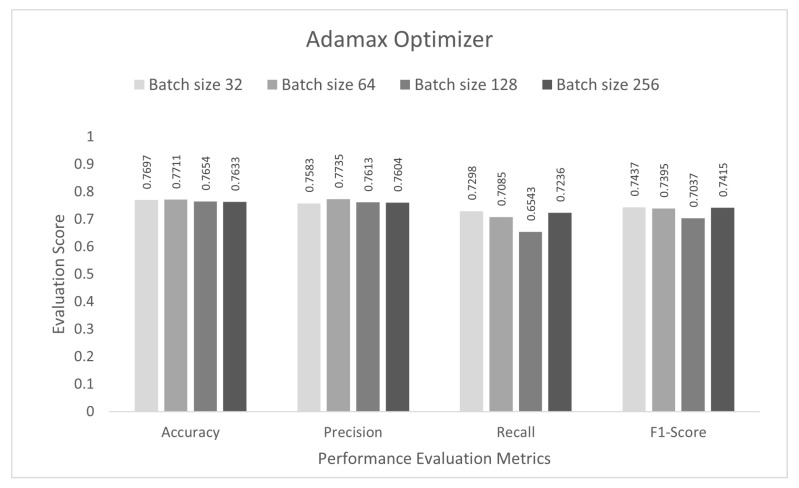
AdaMax optimizer for multi-class sub-category classification scenario.

In the final stage, the performance of the proposed study was evaluated for multi-class subcategory classification scenarios. The DCNN model was trained with the IoTID20 dataset for 100 epochs, and a sparse categorical cross-entropy function was used to calculate the loss. As noted previously, for the binary and multi-class category studies, an Adam optimizer was chosen at the initial stage. The proposed DCNN performance for the Adam optimizer is compared in the bar graphs in Figure 9. Based on the analysis of the results, the proposed model had the highest anomaly detection accuracy of 77.55% at batch size 32. For this optimizer, the other performance scores, namely, precision, recall, and F1-score, were 78.76%, 73.43%, and 76.00%, respectively. In the second step, all the experiments for the Nadam optimizer were rearranged with the same batch sizes. The proposed DCNN performance for the Nadam optimizer is compared in the bar graphs in Figure 10. Based on the analysis of the results, the proposed model had the highest anomaly detection accuracy of 77.44% at batch size 64. For this optimizer, the other performance scores, namely, precision, recall, and F1-score, were 86.02%, 72.58%, and 78.73%, respectively. In the third step, all the experiments for the AdaMax optimizer were repeated with the same batch sizes. The proposed DCNN performance for the Nadam optimizer is compared in the bar graphs in Figure 11. Based on the analysis of the results, the proposed model had the highest anomaly detection accuracy of 77.11% at batch size 64. For this optimizer, the other performance scores, namely, precision, recall, and F1-score, were 77.35%, 70.85%, and 73.95%, respectively.

### 4.4. Performance Discussion

The performance of the proposed DCNN was analyzed for binary, multi-class category, and multi-class subcategory classification. The results presented earlier show a comparison of optimizers and batch sizes. Based on the performance analysis of the proposed model for binary class, the Nadam optimizer with a batch size of 128 performs better than the others. Similarly, in the performance analysis of the proposed model for the multi-class category and subcategory classification, the Adam optimizer with a batch size of 32 performs better than others. For testing the performance of the proposed model, k-fold cross-validation was also used, where the “k” value is 7. The results of the k-fold cross-validation are approximately equivalent.

### 4.5. Performance Comparison with Other DL and Traditional ML-Based IDSs

The performance of the proposed DCNN was compared with other DL and traditional ML methods to evaluate its efficacy. LSTM, gated recurrent unit (GRU), deep neural network (DNN), deep belief network (DBN), deep autoencoder (DAE), and multilayer perceptron (MLP) are examples of DL methods. Decision tree (DT), logistic regression (LR), naive Bayes (NB), support vector machine (SVM), and k-nearest neighbors (KNN) are all examples of traditional ML methods. All of these methods were implemented in the same environment for an accurate performance comparison. The preprocessing steps were the same for all models, including the proposed model. We split the dataset into 80% train and 20% test sets. For all of the DL algorithms, we used Adam optimizer and default batch size 32. The optimal solution of each model was used for the comparison. The hidden layers used in LSTM, GRU, DNN, DBN, AE, and MLP are 3, 3, 4, 4, 6, and 10, respectively. The number of training epochs for all these models was the same as the proposed model. A detailed analysis for binary-class category, multi-class category, and subcategory classifications is shown in Table 8, Table 9 and Table 10, respectively. According to the results, the performance of the proposed DCNN model is optimal as compared to other DL models. The proposed model detection accuracy is 99.84%, 98.12%, and 77.55% for binary-class, multi-class, and subcategory classifications, respectively.

For optimal performance, each DL model requires multiple layers that maximize computational power. The proposed DCNN model improves the performance and also reduces computational power as it narrows to specific features, compared to other ML and DL models. Comparing the performance of the proposed DCNN with other ML and DL models shows the optimal results.

## 5. Conclusions

This study presents a new DCNN-based DL model and feature engineering method for malicious attack detection in IoT networks. The objective was to improve performance and reduce computational power. The proposed DCNN model successfully improves performance and reduces computational power. It is useful for low-power IoT network devices. The IoTID20 dataset was used to analyze the performance of the proposed DCNN model. The proposed model was evaluated for binary, multi-class category, and subcategory classifications. Experiments were performed for different layers of the CNN algorithm, and an optimal solution was selected. The proposed model was evaluated in-depth with Adam, Nadam, and AdaMax optimizers. The Nadam optimizer peformance was optimum for binary, multi-class category, and multi-class subcategory with 128, 32, and 64 batch sizes, respectively. The proposed model was also compared with state-of-the-art DL techniques and other traditional ML algorithms for a broader view in terms of efficacy, robustness, etc. The experimental analysis indicates that the proposed approach obtained optimum results when compared through accuracy, precision, recall, and F1-score parameters.

## Figures and Tables

**Table 1:** A comparison of existing work related to intrusion detection in IoT.

Authors	Year	Technique	Dataset	Multi-Class Detection	Sub-Categories Multi-Class Detection
Basati et al. [19]	2022	DFE	KDDCup99, CICIDS2017, UNSW-NB15	✓	×
Rashid et al. [20]	2022	Ensemble	NSL-KDD, UNSW-NB15	×	×
Fatani et al. [21]	2022	AQU, PSO	CIC2017, NSL-KDD, BoT-IoT, KDD99	✓	×
Alkahtani et al. [22]	2021	CNN-LSTM	IoTID20	×	×
Keserwani et al. [23]	2021	GWO–PSO–RF	KDDCup99, NSL–KDD, CICIDS-2017	✓	×
Qaddoura et al. [24]	2021	SLFN-SVM-SMOTE	IoTID20	✓	×
Saba et al. [25]	2021	GA-(SVM, Ensemble, DT)	NSL-KDD	✓	×
Propose Study	2022	CNN-DNN	IoTID20	✓	✓

**Table 2:** Label details of IoTID20 dataset.

Binary	Category	Subcategory
Normal	Normal	Normal
Anomaly	DoS	DoS-Synflooding
	Mirai	Mirai-Ackflooding
		Mirai-HTTP Flooding
		Mirai-Hostbruteforceg
		Mirai-UDP Flooding
	MITM	MITM ARP Spoofing
	Scan	Scan Port OS
		Scan Hostport

**Table 3:** A detailed distribution of IoTID20 dataset in train and test.

Type	Class	Instances	Train Set	Test Set
Binary	Anomaly	585,342	468,274	117,068
	Normal	40,073	32,058	8015
Total		625,415	500,332	125,083
Category	Mirai	415,309	332,247	83,062
	Scan	75,265	60,212	15,053
	DoS	59,391	47,513	11,878
	MITM ARP Spoofing	35,377	28,302	7075
	Normal	40,073	32,058	8015
Total		625,415	500,332	125,083
Sub-Category	Mirai-UDP Flooding	183,189	146,551	36,638
	Mirai-Hostbruteforceg	121,178	96,943	24,235
	Mirai-HTTP Flooding	55,818	44,654	11,164
	Mirai-Ackflooding	55,124	44,099	11,025
	DoS-Synflooding	59,391	47,513	11,878
	Scan Port OS	53,073	42,458	10,615
	Scan Hostport	22,192	17,754	4438
	MITM ARP Spoofing	35,377	28,302	7075
	Normal	40,073	32,058	8015
Total		625,415	500,332	125,083

**Table 4:** A comparison of CNN layers for multi-class category classification.

Convolutional Layers	Dense Layers	Accuracy	Precision	Recall	F1-Score
1	1	0.9465	0.92	0.9297	0.9237
1	3	0.9798	0.9712	0.9723	0.9716
2	1	0.9791	0.9756	0.9656	0.9701
2	2	0.9823	0.9744	0.9753	0.9747
**2**	**3**	**0.9833**	**0.9742**	**0.9788**	**0.9764**
2	4	0.9794	0.9697	0.9735	0.9713
2	5	0.9813	0.974	0.9757	0.9744

**Table 5:** A comparison of CNN layers for multi-class sub-category classification.

Convolutional Layers	Dense Layers	Accuracy	Precision	Recall	F1-Score
1	1	0.7232	0.7056	0.6443	0.6182
1	3	0.7633	0.7660	0.7157	0.6804
2	1	0.7690	0.7518	0.6563	0.7008
2	2	0.7731	0.7955	0.7320	0.6989
**2**	**3**	**0.7755**	**0.7876**	**0.7343**	**0.7600**
2	4	0.7732	0.7890	0.6790	0.6541
2	5	0.7650	0.8499	0.6527	0.6160

**Table 6:** A detailed comparison of optimizers for multi-class category classification.

Optimizer	Accuracy	Precision	Recall	F1-Score
SGD	0.9789	0.9676	0.9706	0.9690
RMSprop	0.7630	0.7457	0.7195	0.6527
Adam	0.9801	0.9761	0.9695	0.9725
Nadam	0.9838	0.9773	0.9783	0.9777
AdaMax	0.9806	0.9726	0.9721	0.9723

**Table 7:** A detailed comparison of optimizers for multi-class sub-category classification.

Optimizer	Accuracy	Precision	Recall	F1-Score
SGD	0.9789	0.9676	0.9706	0.969
RMSprop	0.7630	0.7457	0.7195	0.6527
Adam	0.9801	0.9761	0.9695	0.9725
Nadam	0.9838	0.9773	0.9783	0.9777
Adamax	0.9806	0.9726	0.9721	0.9723

**Table 8:** A comparison of DCNN with other DL models on binary-class.

Models	Accuracy	Precision	Recall	F1-Score
LSTM	0.9952	0.9943	0.9662	0.9797
GRU	0.9959	0.9856	0.9807	0.9832
DNN	0.9981	0.9983	0.9862	0.9922
DBN	0.9969	0.9937	0.9807	0.9871
AE	0.9974	0.9895	0.9887	0.9891
MLP	0.9972	0.9938	0.9832	0.9884
DT	0.9857	0.9819	0.9861	0.9840
LR	0.9659	0.9034	0.7879	0.8345
NB	0.6504	0.5765	0.8093	0.6733
SVM	0.9744	0.9199	0.8552	0.8844
KNN	0.9983	0.9964	0.9894	0.9929
**Proposed DCNN**	**0.9984**	**0.9967**	**0.9902**	**0.9934**

**Table 9:** A comparison of DCNN with other DL models on multi-class category.

Model	Accuracy	Precision	Recall	F1-Score
LSTM	0.9584	0.9543	0.9201	0.9355
GRU	0.9681	0.9576	0.9468	0.9519
DNN	0.9547	0.9340	0.9447	0.9367
DBN	0.9589	0.9430	0.9549	0.9469
AE	0.9644	0.9515	0.9440	0.9456
MLP	0.9238	0.8933	0.8436	0.8529
DT	0.9770	0.9744	0.9737	0.9741
LR	0.8314	0.7728	0.7297	0.7311
NB	0.6772	0.6628	0.7381	0.6479
SVM	0.8557	0.8416	0.7845	0.7883
KNN	0.9793	0.9746	0.9699	0.9722
**Proposed DCNN**	**0.9812**	**0.9713**	**0.9783**	**0.9746**

**Table 10:** A comparison of DCNN with other DL models on multi-class sub-category.

Model	Accuracy	Precision	Recall	F1-Score
LSTM	0.7141	0.6993	0.5992	0.6453
GRU	0.7615	0.7571	0.6996	0.7272
DNN	0.7483	0.7244	0.6610	0.6912
DBN	0.6888	0.6916	0.6166	0.6519
AE	0.7535	0.7805	0.7016	0.7389
MLP	0.7065	0.7124	0.6263	0.6665
DT	0.7530	0.7508	0.7362	0.7413
LR	0.5481	0.4457	0.4239	0.4142
NB	0.5298	0.4878	0.5032	0.4481
SVM	0.6240	0.4888	0.4741	0.4624
KNN	0.7621	0.7634	0.7477	0.7515
**Proposed DCNN**	**0.7755**	**0.7876**	**0.7343**	**0.7600**

## Data Availability

The publicly available dataset can be found at: https://sites.google.com/view/iot-network-intrusion-dataset/home (accessed on 28 January 2022).
